# Bibliography

**Published:** 1895-05

**Authors:** 


### Bibliography.

TRANSACTIONS OF THE WORLD'S COLUMBIAN DENTAL CONGRESS. Two Volumes. Edited for the General Executive Committee. By A. W. Harlan, A.M., M.D., D.D.S. Assisted by Louis Ottofy, D.D.S., Chicago, Illinois.

The length of time that has elapsed since this Congress was held, now a year and a half, seems to give these transactions something of the character of ancient history, yet when these carefully-prepared volumes are examined, covering fifteen hundred and seventy-nine pages, the question seriously arises, how the editors accomplished their arduous labors so soon? It was certainly an herculean task to arrange, and to finally give to the world the difficult material left them by the great convention. That this has been well done the dental world will, we think, universally acknowledge.

We wish we could say as much of the papers presented. A careful review of these two volumes leaves the same impression that was received at the time the Congress was held, and for all practical purposes the tremendous task the *Dental Cosmos* imposed upon itself was as well done and gave as good a general idea of the work performed as these bulky volumes can possibly give.

There is much in them of lasting value, some papers that will live, many that should never have been there, and in one instance, at least, one that should have been of deep interest if properly prepared is left out entirely. Allusion is here made to the history of dentistry. The editors propose that this should be published in a separate volume. This may be the best course, but unless published by individual aid it is not likely to reach library shelves. As a matter of dental history, if for no other reason, those not supplied with these transactions should send early and secure copies, as the supply is limited.

DENTAL MEDICINE: A MANUAL OF DENTAL MATERIA MEDICA AND THERAPEUTICS. By Ferdinand J. S. Gorgas, A.M., M.D., D.D.S. Fifth Edition, Revised and Enlarged. P. Blakiston, Son & Co., Philadelphia, 1895.

This work of Professor Gorgas is so well established in reputation as a text-book in dental colleges that it would seem superfluous to do more than announce the rapid sale indicated by the present issue.

It was very unfortunate that the author or publisher permitted the fourth edition to run out just previous to the opening of the college term without a reissue, and now it is too late to be of much service this year.

While the number of books on materia medica are legion, no one of them is exactly fitted to meet the needs of the dental student except this of Dr. Gorgas. In expressing this it is not to be understood that it is regarded as perfection.

Exactly why the author has persistently failed to notice criticisms on previous editions may be best answered by himself or the publisher, but it certainly is not to the credit of some one that "Chas." Truman still remains on page 175, when special attention was called to it in a former review.

The author is not justified in continuing the paragraph on page 178, beginning, "Arsenious acid is also employed in dental practice to obtund the undue sensitiveness," etc. If this is to continue as part of the text, colleges should refuse to longer accept it, for certainly it is not to be credited that any respectable practitioner makes use of this agent for this purpose.

The paragraph on the "dental uses of aconite" still remains as it was in other editions. The author is entitled to his views on this subject, but to the writer they seem very erroneous.

In the use of nitrate of silver, no allusion is made to Dr. Stebbins's valuable and original suggestions for its use in deciduous teeth, but in place thereof he gives Dr. Peirce's use of blotting-paper saturated with the agent. Now, as the preparation of the latter was based on Dr. Stebbins's suggestions, it is difficult to see why proper credit has not been given.

The author still continues the imperfect statement regarding the use of chlorinated lime in bleaching teeth, page 287, and gives no credit to the author of the process.

No mention is made of the use of quinine in local treatment of gingivitis, pyorrhœa, etc., yet its use has been before the profession for several years.

We regret to be obliged to call attention to these specimens of careless editing, but neither authors nor publishers have a right to issue and reissue defective publications after attention has been called thereto.

The book has been enlarged by the addition of a number of recently introduced agents.

CATCHING'S COMPENDIUM OF PRACTICAL DENTISTRY FOR 1894. By B. H. Catching, D.D.S., Editor and Publisher, Atlanta, Georgia.

To close up the work of the year 1894 and issue this book of three hundred pages early in 1895 must have been a labor that strained the energy of the editor, great as that is known to be. It is doubtful whether the work connected with abstracting the pith of articles in the numerous journals in this country and Europe is appreciated. The book must be entirely written out, and to do this intelligently requires an ability of a peculiar order. This the editor, Dr. Catching, certainly possesses.

The compendium has become a necessary addition to the dental library, for by its aid the busy operator can see at a glance the important work of the year condensed for practical use.

It is a satisfaction to find that the publisher has adopted the suggestion of this journal and secured efficient aid from foreign countries. In time this will have a greater development than is manifested in this volume, and it is hoped it will eventually become the depository of the yearly work of the world in dentistry. To accomplish this Dr. Catching needs and should receive the cordial and earnest assistance of professional men everywhere. This can best be accomplished by procuring the compendium and thus securing a large circulation.

Descriptive Anatomy of the Human Teeth. Third edition. By G. V. Black, M.D., D.D.S., Sc.D. Published by The Wilmington Dental Manufacturing Company, Philadelphia.

It seems scarcely necessary to do more than refer to any book having Dr. Black's name as the author, and this is especially true with this valuable production from his pen. That it has filled an important place in the instruction of students is evident from the fact that the first edition was issued in 1891, and now at the begining of 1895 the third is demanded.

The illustrations are not only satisfactory as representations of correct forms, but they are arranged in the best possible way for study.

Dr. Black in this edition has made but few changes in the nomenclature, evidently feeling that it is wise to move slowly in this direction. "The changes . . . consist of one new word, *axial*, two new word forms, *occlusal* in place of *occluding*, and *incisal* in compound forms."

The author deserves and should receive the credit of having through this book inspired a wider interest and more exact study of tooth forms than was thought necessary in the earlier dental education. For this and much other faithful work the dentists of the country owe him a large debt of gratitude.

